# Variation in Fatty Acid Distribution of Different Acyl Lipids in Rice (*Oryza sativa* L.) Brans

**DOI:** 10.3390/nu3040505

**Published:** 2011-04-21

**Authors:** Hiromi Yoshida, Takaaki Tanigawa, Isoko Kuriyama, Naoko Yoshida, Yuka Tomiyama, Yoshiyuki Mizushina

**Affiliations:** 1 Department of Nutritional Science, Kobe Gakuin University, Kobe, Hyogo 651-2180, Japan; Email: nhaob016@s.kobegakuin.ac.jp (T.T.); kuriyama@nutr.kobegakuin.ac.jp (I.K.); tomiyama@nutr.kobegakuin.ac.jp (Y.T.); mizushina@nutr.kobegakuin.ac.jp (Y.M.); 2 Cooperative Research Center of Life Sciences, Kobe Gakuin University, Kobe, Hyogo 650-8586, Japan; 3 Department of Biomedical Sciences, College of Life Sciences, Ritsumeikan University, Kusatsu, Shiga 525-8577, Japan; Email: nyoshida@sk.ritsumei.ac.jp

**Keywords:** different acyl lipids, fatty acid distributions, phospholipids, rice bran lipids, triacylglycerols

## Abstract

The lipids extracted from rice brans were classified by thin-layer chromatography into eight fractions, and their fatty acid (FA) compositions were investigated among five different Japanese cultivars. The lipids of these rice brans comprised mainly triacylglycerols (TAG; 84.9-86.0 wt%), free FA (4.2-4.6 wt%), and phospholipids (PL; 6.5-6.7 wt%), whilst other components were also detected in minor proportions (0.2-2.1 wt%). The PL components included phosphatidyl choline (43.3-46.8 wt%) phosphatidyl ethanolamine (25.0-27.3 wt%) and phosphatidyl inositol (20.2-23.2 wt%). Comparison of the different cultivars showed, with a few exceptions, no substantial difference (*P* > 0.05) in FA distribution. FA distribution of TAG among the five cultivars was characterized as: unsaturated FA predominantly concentrated at the *sn*-2 position and saturated FA primarily occupying the *sn*-1 or *sn*-3 position in these lipids. These results suggest that the rice bran lipids may be well incorporated into our daily diet to improve nutritional value of the Japanese diet.

## 1. Introduction

Rice (*Oryza sativa* L.) is one of the most important cereal crops cultivated in the world; it feeds more than half of the world’s population [1]. Rice is the staple food in Japan and other East/Southeast Asian countries. Japan is self-sufficient in rice. Rice grain quality is an important economic trait that influences rice production in many rice-producing areas. Although the fat or oil in rice grain is low (*i.e.*, 2-3%) and is concentrated in the germ and bran fractions, it is a key determinant of the processing and cooking quality of rice [2]. For instance, the surface lipid content has been thought to be an indication of the degree of milling [3]. In addition, rice lipid, frequently forming complexes with starch granules [4], was shown to affect starch gelatinization, water availability to starch, and rice swelling and thus influenced rice eating and cooking quality [5]. Rice brans also contain a number of nonfiber constituents in the nonsaponifiable oil fraction that have been implicated as factors in lowering cholesterol, including oryzanol [6].

Besides dietary consumption, the unique health benefits of rice fat, which includes many unsaturated fatty acids, have drawn much attention [7]. A number of studies have shown that rice bran oil reduces the harmful cholesterol (LDL) without changing good cholesterol (HDL) [8]. On the other hand, some reports showed that the hydrolysis and oxidation of rice fat are responsible for rice aging and deterioration of grain flavor during storage, and low-oil rice cultivars are more suitable for grain storage [9]. Although many extensive studies have been made on rice bran oil, little is known regarding the rice bran lipids including complex lipids. Some of these lipids are thought to be associated with protein in the native state, particularly in germ, but concrete evidence has yet to be presented. 

To the best of our knowledge, many investigations on lipid fractions of rice brans have been published. However, research dealing with a comparison study between different rice bran cultivars is limited. Therefore, the aim of the present study is to compare the lipid components and fatty acid (FA) distribution of different acyl lipids obtained from five different rice bran cultivars. 

## 2. Experimental Section

### 2.1. Rice Seeds

Commercially obtained mature rice seeds (*Oryza sativa* var *japonica*) used in this study were from five different Japanese cultivars; *Koshihikari*, *Haenuki*, *Akitakomachi*, *Hitomibore* and *Sasanishiki*. These seeds were harvested at Akita prefecture in Japan on September of 2009. These seeds were hand-selected to eliminate those that were cracked or otherwise damaged and then packed in polyethylene bags under nitrogen gas and stored at −20 °C until further analysis.

### 2.2. Reagents and Standards

All chemicals and solvents used were of analytical grade (Nacalai Tesque, Kyoto, Japan), but diethyl ether was further purified to remove peroxides. TLC plates (silica gel 60 G, 20 × 20 cm, 0.25 mm thickness) were obtained from Merck (Darmstadt, Germany). The TLC standard mixture, containing monoacylglycerols (MAG), diacylglycerols (DAG), free fatty acids (FFA), triacylglycerols (TAG), steryl esters (SE) and hydrocarbons (HC), was purchased from Nacalai Tesque (Kyoto, Japan). A phospholipid kit from Serdary Research Laboratory (Mississauga, ONT, Canada) was used as phospholipid standard. Lipase from porcine pancreas was obtained from Sigma Chemical Co. (St. Louis, MO, USA), and used after purification with acetone and then diethyl ether as described previously [10]. Glyceryl-*sn*-1,3-myristate-*sn*-2-oleate (Sigma Chemical Co.) was used as TAG standard for enzymatic hydrolysis. FA methyl ester (FAME) standards (F & OR mixture #3) were obtained from Altech-Applied Science (State College, PA, USA). Methyl pentadecanoate (15:0, 100 mg; Merck, Darmstadt, Germany) was dissolved in *n*-hexane (20 mL) and used as the internal standard. Boron trifluoride (BF_3_) in methanol (14%; Wako Pure Chemical Inc., Osaka, Japan) was used to prepare FAME.

### 2.3. Extraction of Lipids

Rice of each cultivar (1 kg) was milled by a domestic miller (Zojirushi Ltd BR-CA25 Osaka, Japan); such that rice brans were well-milled before extraction. The total lipids were extracted from the brans (200 g) for 10 min at 0 °C with 300 mL chloroform/methanol (2:1, v/v), following the Folch procedure [11]. These solvents contained 0.01% butylated hydroxytoluene to inhibit the oxidative degradation of lipids during analysis. Extraction was repeated three times, then 20 mL aqueous KCl (0.75%) was added to the combined extracts. After phase separation, the chloroform layer was withdrawn, dried over anhydrous Na_2_SO_4_, filtered, and the filtrate was concentrated under vacuum in a rotary evaporator at 35 °C. The extracted lipids were weighed to determine the lipid content of the rice brans and then transferred to a 25 mL brown-glass volumetric flask with chloroform/methanol (2:1, v/v).

### 2.4. Lipid Analysis

Using a previously described method [12], total lipids were fractionated by TLC into eight subfractions with *n*-hexane/diethyl ether/acetic acid (80:20:1, v/v/v). Bands corresponding to HC, SE, TAG, unknown, FFA, 1,3-DAG, 1,2-DAG and phospholipids (PL) were scraped into test-tubes (105 × 16 mm; poly (tetrafluoroethylene)-coated screw caps). Methyl pentadecanoate (10-100 μg) from a standard solution (5 mg/mL) as the internal standard was added to each tube with a microsyringe (Hamilton Co., Reno, NV, USA). With the exception of HC, FAME were prepared from the isolated lipids by heating with silica-gel for 30 min at 80 °C in BF_3_/methanol (3 mL) on an aluminum block bath [13]. Following a previously reported method [14], the *n*-hexane layer containing the FAME was recovered and dried over anhydrous Na_2_SO_4_. The solvent was then vaporized under a gentle stream of nitrogen, and the residue (FAME) was quantified by gas chromatography (GC) using a Shimadzu Model-14B GC (Shimadzu, Kyoto, Japan) equipped with a hydrogen flame ionization detector (FID); a polar capillary column (ULBO HE-SS-10 for FAME fused silica WCOT (serial No. PSC5481); cyanopropyl silicone, 30 m × 0.32 mm i.d. (Shinwa Chem. Ind., Ltd., Kyoto, Japan).

Helium was used as the carrier gas, at a flow rate of 1.5 mL/min, and the GC was operated under a constant pressure of 180 kPa. The oven temperature was programmed from an initial temperature of 180 °C (held for 2 min), rising to 200 °C at a rate of 2 °C/min, and then held isothermally (200 °C) for 15 min. Both injection and detector temperatures were set at 250 °C. All samples were dissolved in *n*-hexane for injection. FA was identified by comparison of the retention times with those of standard FAME and the results are reported as a weight percentage of the lipid. The other GC conditions were as previously described [15]. 

Samples of the extracted polar lipids, which had been separated, were further isolated by TLC into several fractions with chloroform/methanol/acetic acid/deionized water (170:30:20:7, by volume) as the mobile phase. PL classes were detected by iodine vapor and were consistent with the authentic standards. Bands corresponding to PE, PC, PI and others were carefully scraped off into test tubes. Then, FAME were prepared by the same method as described above and analyzed by GC.

### 2.5. Enzymatic Hydrolysis of Lipids

TAG hydrolysis was carried out *in vitro* as previously reported [10]. A 30 min reaction was selected based on the preliminary results using the standard TAG (glyceryl-*sn*-1,3-myristate-*sn*-2-oleate: Sigma Chemical Co.). After approximately 60% of TAG was hydrolyzed, 0.5 mL 6 M HCl and 1 mL ethanol were added to stop the reaction. In the preliminary study, no FA (oleic acid) at the *sn*-2 position of standard TAG was transferred to the *sn*-1 or *sn*-3 position at 60% hydrolysis for 30 min. The reaction products were separated by TLC as previously reported [10]. The FFA and *sn*-2 MAG bands were carefully scraped off into test tubes and then methylated [13]. The procedure was checked by comparing the FA compositions of the original TAG and the TAG remaining after partial hydrolysis.

### 2.6. Statistical Analyses

All preparations and determinations were carried out in triplicate, and the results were subjected to one-way analysis of variance (ANOVA) [16]. Significant differences (*P* < 0.05) were calculated using multiple comparison tests, following a previously reported method [17].

## 3. Results and Discussion

### 3.1. Lipid Components in the Rice Brans

**Figure 1:**
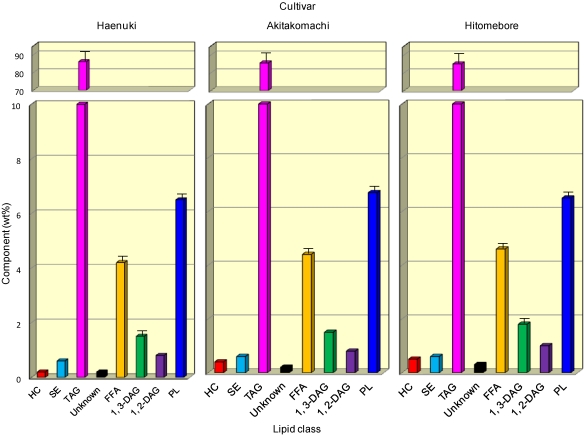
Lipid components in the oils obtained from rice brans of three Japanese cultivars. Each value represents the average of three determinations, and vertical bars depict the mean and standard deviation. HC, hydrocarbons; SE, steryl esters; TAG, triacylglycerols; FFA, free fatty acids; DAG, diacylglycerols; PL, phospholipids.

The compositional analyses carried out in this work included determination of the lipid classes and the FA compositions of the lipids. For all of the five cultivars, the original amount of total lipids was a range of 4435-4585 mg per 20 g brans. Therefore, no substantial difference (*P* > 0.05) in content of total lipids could be observed among all five cultivars. Profiles of the different lipid classes in the rice brans are shown in Figure 1; the data for *Koshihikari* and *Sasanishiki* were omitted as their patterns were very similar to those of *Haenuki*, *Akitakomachi* and *Hitomebore*. Predominant components were TAG (84.9-86.0 wt%), followed by free FA (FFA; 4.2-4.6 wt%) and PL (6.5-6.7 wt%) and minute amounts of additional components (0.2-2.1 wt%). When comparing the eight lipid components among all five cultivars, no substantial difference (*P* > 0.05) in the content of the lipid components could be observed using values estimated by combining thin-layer chromatography (TLC) and gas chromatography (GC) using the internal standard (15:0). Presumably, the minor components, such as FFA, 1,3- and 1,2-DAG, may be due to the partial enzymatic hydrolysis of reserve TAG during storage of the rice seeds [18]. The lipid components resulting from “fat by hydrolysis” in starch granules were examined, showing the presence of FFA with lysolecithin and lysoglycolipids [19].

### 3.2. Lipid Components of Major Phospholipids in the Rice Brans

**Figure 2:**
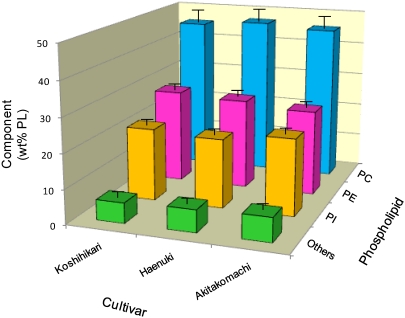
PL components in the oils prepared from rice brans. Each value represents the average of three determinations, and vertical bars depict the mean and standard deviation. “Others” include minor PL components such as diphosphatidyl glycerol, phosphatidic acid, phosphatidyl glycerol, lysophospholipids and lysoglycolipids. PE, phosphatidyl ethanol-amine; PC, phosphatidyl choline; PI, phosphatidyl inositol.

To clarify the distribution of individual PL in the rice brans, further separation of the PL fraction into several fractions, such as phosphatidyl ethanolamine (PE), phosphatidyl choline (PC), phosphatidyl inositol (PI) and others was carried out on TLC in the presence of authentic standards. Other PL included diphosphatidyl glycerol, phosphatidic acid, phosphatidyl glycerol, lysophospho-lipids and lysoglycolipids. Comparisons were made between the profiles of PE, PC, PI and the others of all five cultivars (Figure 2); the data for *Hitomebore* and *Sasanishiki* were omitted as their patterns were very similar to those of *Koshihikari*, *Haenuki* and *Akitakomachi*. For all the five cultivars, the original amounts of each PL were in a range of 129-137 mg (43.3-46.8 wt%), 76-81 mg (25.0-27.3 wt%) and 58.2-69.1 mg (20.2-23.2 wt%) per 20 g brans for PC, PE and PI, respectively. These PL are known to be essential components of the cell membranes in plants. Since membrane lipids are involved in such fundamental cell processes as ion transport, energy generation and biological reactions, they are highly conserved in terms of both quality and quantity [20]. 

### 3.3. FA Composition of Major Lipids in the Rice Brans

**Figure 3:**
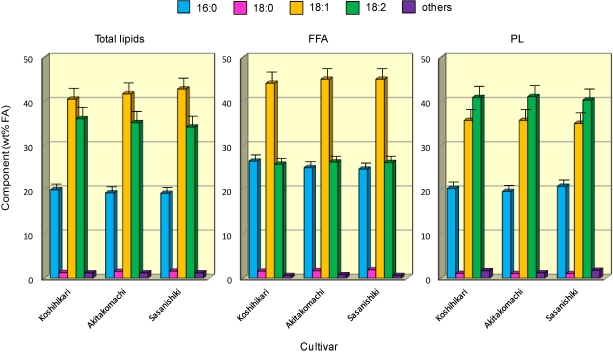
FA distribution in total lipids (left panel), FFA (middle) and PL (right) prepared from rice brans. Each value represents the average of three determinations, and vertical bars depict the mean and standard deviation. “Others” include minor FA such as 14:0, 16:1, 18:3 and 20:0. For abbreviations, see Figure 1.

**Figure 4:**
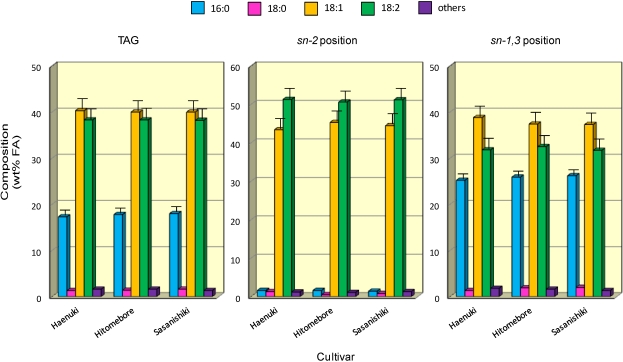
Composition and positional distribution of FA in TAG prepared from rice brans. Each value represents the average of three determinations, and vertical bars depict the mean and standard deviation. “Others” include minor FA such as 14:0, 16:1, 18:3 and 20:0. For abbreviations, see Figure 1.

FA composition (expressed in terms of the esters by weight) of total lipids, FFA and PL in the rice brans were compared among all five cultivars (Figure 3); the data for *Haenuki* and *Hitomebore* were omitted as their patterns were very similar to those of *Koshihikari*, *Akitakomachi* and *Sasanishiki*. The principal FA components are generally palmitic (16:0), oleic (18:1*n*-9) and linoleic (18:2*n*-6) acids, the distribution of which differs according to these lipid classes. The samples presented significant amounts of total unsaturated FA, which consisted mainly of oleic (18:1*n*-9) acid followed by linoleic (18:2*n*-6) acid, in the total lipids, FFA and TAG (Figure 4), whilst the reverse was true for the FA distribution of PL, representing 78.1-79.3 wt%, 79.8-80.3 wt%, 71.1-73.2 wt% and 77.5-78.8 wt% for total lipids, TAG, FFA and PL, respectively. 

Some differences (*P* < 0.05) in FA composition were noted when the four lipid classes were compared (Figure 3 and ). With a few exceptions, the percentage of palmitic (16:0) acid was significantly (*P* < 0.05) higher in the FFA, whilst that of linoleic (18:2*n*-6) acid was higher (*P* < 0.05) in the PL (Figure 3). The percentage of oleic (18:1) acid was significantly (*P* < 0.05) higher than linoleic (18:2*n*-6) acid in the total lipids, TAG and FFA fractions. The data for FA distribution of minor lipid components, such as SE 1,3- and 1,2-DAG (Figure 1), were not included in Figure 3 as these lipid components were too small to obtain reliable results for these lipids. 

### 3.4. FA Positional Distribution of TAG in the Rice Brans

The characteristics of the components and positional distribution of FA in the TAG were compared between all five cultivars (Figure 4); the data for *Koshihikari* and *Akitakomachi* were omitted as their patterns were very similar to those of *Haenuki*, *Hitomebore* and *Sasanishiki*. Linoleic (18:2*n*-6) was predominantly (50.2-52.8 wt%) concentrated in the *sn*-2 position of the TAG molecules, whilst saturated FA such as palmitic (16:0) and steraric (18:0) acids primarily occupied the *sn*-1 or *sn*-3 position. With a few exceptions, however, oleic acid (18:1*n*-9) was almost evenly distributed in the *sn*-1, 2 or 3 position, as other researchers also reported [21]. No significant difference (*P* > 0.05) occurred in the FA distributions among all five cultivars. Taken together, the regiospecific distribution profiles for the FA of TAG were very similar to the results obtained from other plant seed lipids such as soybeans, corn [22] or broad beans [15]. 

### 3.5. FA Compositions of Major Phospholipids in the Rice Brans

**Figure 5:**
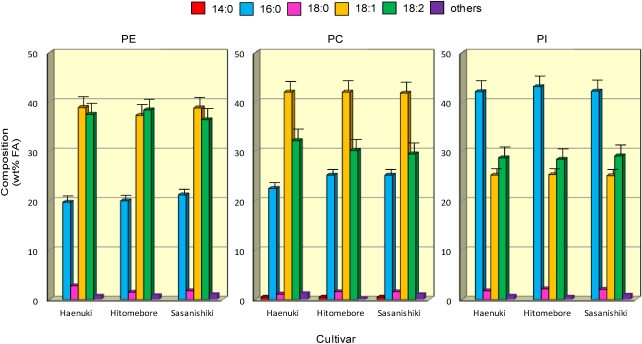
FA profiles for the major PL (PE, left panel; PC, middle panel; and PI, right panel) prepared from rice brans. Each value represents the average of three replicates, and vertical bars depict the mean and standard deviation. “Others” include minor FA such as 14:0, 16:1, 18:3 and 20:0. For abbreviations, see Figure 1 and Figure 2.

Figure 5 shows typical FA distributions for the PE, PC and PI fractions among all five cultivars; the data for *Koshihikari* and *Akitakomachi* were omitted as their patterns were very similar to those of *Haenuki*, *Hitomebore* and *Sasanishiki*. The major FA in the three PL were commonly palmitic (16:0), oleic (18:1*n*-9) and linoleic (18:2*n*-6) acids. The data showed that the percentage composition of linoleic (18:2*n*-6) was higher in PE than in PC, whilst that of oleic (18:1*n*-9) acid was higher in PC than in PE. These FA distribution patterns seen for PE and PC were very similar among all five cultivars: palmitic (19.7-25.2 wt%), oleic (38.8-43.6 wt%) and linoleic (29.5-37.5 wt%). However, PI was unique in that it had the highest saturated FA (43.9-45.6 wt%) content among the three PL, and the distribution pattern was very similar among all five cultivars. Particularly, the percentage of palmitic (16:0) acid was significantly (*P* < 0.05) higher in PI (42.1-42.5 wt%) than in PE (18.9-21.2 wt%) or PC (22.4-25.2 wt%) among all five cultivars. The data for FA distributions of minor lipid components, such as diphosphatidyl glycerol, phosphatidic acid, phosphatidyl glycerol, lysophospho-lipids and lysophosphoglycolipids, were omitted from Figure 5 because these PL components could not be isolated perfectly from each other. Therefore, we would like to consider them in our future work.

## 4. Conclusions

The major lipid components in rice brans of the five different Japanese cultivars were TAG, FFA and PL, whilst HC, SE, 1,2- and 1,3-DAG were also present in minor proportions. The PL components included PC, PE and PI. In general, with a few exceptions, no significant difference (*P* > 0.05) was observed in the FA distribution patterns among all five cultivars. FA distribution of TAG among all five cultivars was characterized as: unsaturated FA predominantly concentrated at the *sn*-2 position and saturated FA primary occupying the *sn*-1 or *sn*-3 position in these lipids. The distribution patterns in the different acyl lipids and their FA profiles in rice brans were very similar to each other among the five cultivars. Therefore, the lipid composition suggests that these rice bran lipids could be a good source of nutraceuticals with positive health benefits. The rice bran lipids may be well incorporated into our daily diet to improve the nutritional value of the Japanese diet.
